# Tissue-Specific Accumulation and Dietary Risk of Arsenic and Other Potentially Toxic Elements in Retail Meats

**DOI:** 10.3390/jox16030090

**Published:** 2026-05-21

**Authors:** Syed Sayyam Abbas, Syed Ali Musstjab Akber Shah Eqani, Ismat Nawaz, Mansoor A. Alghamdi, Ahmed S. Summan, Abdul Qadir, Shabbar Abbas, Iqra Rasheed, Syeda Maria Ali, Mustafa Nawaz Shafqat, Mohammed I. Orif, Heqing Shen, Nadeem Ali

**Affiliations:** 1 Department of Biosciences, COMSATS University, Islamabad 45550, Pakistan; sayyam33@gmail.com (S.S.A.); alishah@comsats.edu.pk (S.A.M.A.S.E.); shabbar.abbas@comsats.edu.pk (S.A.); mnshafqat@comsats.edu.pk (M.N.S.); 2 Department of Environment, Faculty of Environmental Sciences, King Abdulaziz University, Jeddah 21589, Saudi Arabia; mghamdi2@kau.edu.sa (M.A.A.); asumman@kau.edu.sa (A.S.S.); 3 Center of Excellence in Environmental Sciences, Kind Abdul Aziz University, Jeddah 21589, Saudi Arabia; morif99@yahoo.com; 4 College of Earth and Environmental Sciences, University of the Punjab, Lahore, 54590, Pakistan; aqfics@yahoo.com; 5 Department of Environmental Sciences, International Islamic University, Islamabad, 44000, Pakistan; iqraarasheed.17@gmail.com (I.R.); maria.ali@iiu.edu.pk (S.M.A.); 6 Department of Marine Chemistry, Faculty of Marine Sciences, King Abdulaziz University, Jeddah 21589, Saudi Arabia; 7 Institute of Urban Environment, Chinese Academy of Sciences, Xiamen 361021, China; hqshen@xmu.edu.cn

**Keywords:** PTEs, meat contamination, risk assessment, food safety, Pakistan

## Abstract

Data on arsenic (As) and other potentially toxic elements (PTEs) in Pakistani retail meats are limited, constraining evidence-based dietary risk assessment and management. This study aimed to determine the concentrations and profiles of As and seven other PTEs (Cr, Ni, Mn, Pb, Cd, Cu, Zn) in commonly consumed meats and to evaluate the associated non-carcinogenic health risks. Ninety-two paired liver and muscle samples from broiler chicken, goat (mutton), and beef cattle were collected from four cities across the Indus Plain and analyzed using inductively coupled plasma mass spectrometry (ICP-MS). Dietary exposure was evaluated using estimated daily intake (EDI), target hazard quotient (THQ), and hazardous index (HI) under typical and high-consumption scenarios. Overall, Zn and Cu exhibited the highest concentrations, followed by Mn and Cr, whereas As, Pb, Ni, and Cd occurred at comparatively lower but environmentally relevant levels. Beef liver exhibited the highest contamination levels, exceeding FAO/WHO permissible limits for Pb, Cu, and Cd in up to 40% of samples. In contrast, mutton and beef muscle contained the highest As and Zn concentrations, while chicken muscle showed elevated Cr levels. Multivariate statistical analysis revealed three dominant co-variation patterns, suggesting potential contamination pathways: (i) geogenic groundwater sources enriched with As, Cr, and Ni; (ii) atmospheric and industrial dust inputs linked with Pb, Cd, and Mn; (iii) mineral-enriched feed additives potentially contributing to elevated Zn and Cu, particularly in poultry. Under high-consumption scenarios, THQ values for As, Cr, Cu, and Zn exceeded the safety threshold (THQ > 1), highlighting beef products as the dominant source of chronic dietary risk. Overall, the findings highlight pronounced tissue- and species-specific accumulation trends, and emphasizes the urgent need for stricter feed and water quality control measures to minimize dietary exposure to PTEs.

## 1. Introduction

In developing countries, both geogenic processes and extensive anthropogenic activities have contributed to the accumulation of potentially toxic elements (PTEs) in topsoil, groundwater, and aquatic ecosystems over the past two decades, emerging as a major environmental and public health concern [1,2,3,4]. Several studies conducted in Pakistan have reported considerable concentrations of arsenic (As), lead (Pb), and other PTEs in various environmental compartments, including vegetables [5,6], soil [3], and drinking water resources [7,8,9]. Elevated levels of PTEs and As in water and soil systems may cause secondary food chain contamination through bioaccumulation, either directly via drinking water or indirectly through consumption of contaminated plants and small animals.

Livestock exposure to PTEs through the soil–plant system [10,11] and contaminated drinking water increases the likelihood of metal accumulation in edible tissues, posing significant health risks not only to animals but also to humans consuming such meat. This contamination pathway represents a substantial threat to public health in Pakistan [12,13,14]. Globally, numerous studies have assessed PTE contamination in animal tissues such as the liver and muscle [15,16,17,18]. These studies have reported noteworthy concentrations of PTEs, suggesting low-to-severe health risks for animals and their meat consumers across regions including China, the Middle East, Africa, and Pakistan. Moreover, organ-specific (liver vs. muscle) and species-specific (chicken, goat, beef) distributions of toxic elements have been documented [15,16]. Such tissue- and species-based datasets are essential for consumers and regulatory authorities, enabling the development of targeted food safety policies and prioritization of risk mitigation at both national and regional levels [17,19].

With a population nearing 233 million, Pakistan’s annual per capita meat consumption is approximately 32 kg, primarily comprising chicken, beef, and mutton [20]. In such a high-consumption context, the detection of PTEs—particularly Pb, Hg, Cd, Cu, and As—in meat constitutes a serious public-health concern [21]. Chronic dietary exposure to these elements has been associated with renal, hepatic dysfunction, neurological, and carcinogenic outcomes [4,22].

Despite these concerns, systematic surveillance of PTEs in Pakistani meat remains limited. The few available studies—focused on poultry [15,23,24] and ruminant meat [25,26]—were conducted at smaller spatial scales, often using lower-resolution analytical techniques, and rarely translated concentration data into quantitative health risk metrics. Consequently, robust, large-scale evidence on issue- and species-specific accumulation patterns remains scarce. Accordingly, the objectives of this study were to: (i) quantify the concentrations of arsenic and other PTEs in the paired liver and muscle tissues of chicken, mutton, and beef collected from multiple urban centers; (ii) examine tissue- and species-specific accumulation patterns; (iii) explore potential contamination pathways using multivariate statistical approaches; (iv) assess dietary exposure and associated non-carcinogenic health risks using EDI, THQ, and cumulative HI frameworks. Compared with previous localized studies, this investigation provides the first integrated multi-city assessment of paired liver and muscle tissues across major meat types, coupled with multivariate statistical analysis and cumulative dietary risk modeling, thereby offering a more comprehensive and policy-relevant evaluation of PTE exposure through meat consumption.

## 2. Materials and Methods

### 2.1. Study Area

**Figure 1:**
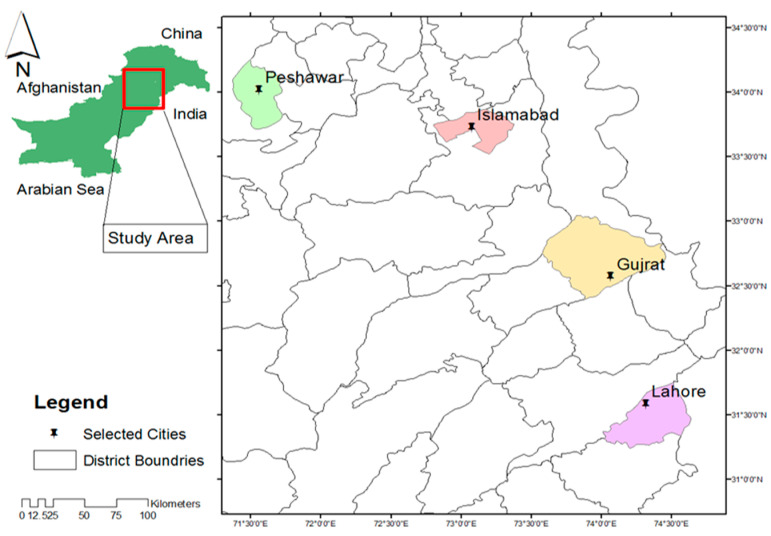
Geographical location of the study area in Pakistan, showing the four selected sampling cities (Peshawar, Islamabad, Gujrat, and Lahore) highlighted in different colors. The inset map indicates the national location of the study region, while district boundaries and sampling points are also illustrated.

The present study was carried out in four different cities of Pakistan, namely Peshawar, Islamabad, Gujrat, and Lahore (Figure 1), for PTE assessment in different types of meat samples (chicken, goat, and beef). Previous studies reported extensive PTE contamination in the drinking water, dust/soil, and food commodities belonging to the areas selected for this study [3,17,27].

Islamabad, located at the foothills of the Margalla range, is primarily influenced by geogenic enrichment of trace elements in soils and aquifers, coupled with rapid urban expansion, vehicular emissions, and construction-related activities, which collectively contribute to atmospheric deposition and diffuse environmental metal loading. Peshawar, situated in the north-western region near the Afghan border, is characterized by intensive industrial activities, brick kilns, solid-waste burning, and domestic fuel combustion, which represent major sources of atmospheric and soil contamination by PTEs. Lahore, a major industrial and commercial hub, is characterized by extensive industrial activities, scrap yards, e-waste recycling facilities, and widespread wastewater irrigation in per-urban agricultural zones, as well as arsenic-contaminated aquifers, which collectively promote PTE accumulation in livestock production systems. Gujrat, situated along the Chenab River, is impacted by industrial discharges, urban runoff, and deteriorating groundwater quality, resulting in elevated PTE levels in surface and subsurface waters. These cities were chosen based on documented environmental contamination, rapid urbanization, industrial emissions, wastewater irrigation practices, and geogenic enrichment of trace elements, which collectively represent major pathways for PTE transfer into the food chain.

### 2.2. Sample Collection and Preparation

Ninety-two paired fresh samples of the muscle and liver of chicken, mutton and beef were collected from the different slaughterhouses and poultry farms of the respective cities during 2022–2023 (Figure 1). Liver and muscle were selected because the liver reflects metal metabolism and accumulation, whereas muscle represents the primary pathway for human dietary exposure. The weight of each sample ranged between 150 and 250 g. After collection, the samples were immediately preserved in an ice box and transferred to the laboratory, where they were classified, weighed, and kept frozen at −20 °C until further analysis. All the samples were collected from the market, and background details about the meat supply system, along with relevant information, were recorded from the shopkeepers. In general, the chicken meat supply is associated with farms (broiler chicken) within the premises of each studied city, whereas goat and beef meat were supplied from nearby rural areas of the respective districts. The information regarding the consumption rate of each meat type was also collected from the local population of all the areas studied. Sampling locations were selected to represent major urban meat supply sources across the study cities and to capture variability in retail supply chains. Because sampling was market-based rather than formal probabilistic random sampling, the dataset should be interpreted as providing screening-level representation of contamination patterns in retail meats.

Prior to analysis, all collected samples were thawed and washed with distilled water. The samples were cleaned by removing excess fats and then chopped into small pieces using a knife. All the samples were then oven-dried at 60 °C for approximately 24 h. After drying, the samples were weighed to determine their moisture contents (% age) and then ground into powders and separated into 10 g for each stored sample. All elemental concentrations were expressed on a dry-weight (dw) basis to minimize analytical variability associated with heterogeneous tissue moisture content and to ensure consistency and comparability with previously published food contamination studies. Since regulatory limits such as FAO/WHO guideline values are commonly expressed on a fresh-weight basis, this difference should be considered when making direct regulatory comparisons. Although dry-weight reporting improves analytical precision and minimizes variability associated with tissue moisture, dietary exposure assessments expressed on a dry-weight basis may overestimate exposure relative to fresh-weight scenarios. Because tissue moisture contents were measured, tissue-specific dry- to fresh-weight conversion can in principle be applied; however, the present study retained dry-weight concentrations as a conservative screening-level approach. We acknowledge that use of fresh-weight equivalents could reduce exposure estimates and improve direct regulatory comparability.

For clarity, Mn, Cu, and Zn are reported in mg/kg, whereas As, Cd, Cr, Ni, and Pb are reported in µg/kg; this reporting convention was applied consistently throughout the manuscript, and unit conversions were performed where required for risk calculations and comparison with guideline values. The samples were sealed in glass vials labeled with an identification code and preserved for digestion.

### 2.3. PTE Analysis

Prior to sample digestion, all containers were soaked in 10% (*v*/*v*) HNO_3_ overnight and rinsed three times with ultrapure water obtained from a Milli-Q system (Millipore Corporation, Billerica, MA, USA). Each sample was carefully weighed (approximately 0.2 g) and digested with 2 mL of GR-grade 65% (*v*/*v*) HNO_3_ (CNW Corporation, Shanghai, China) overnight, followed by 1 mL of GR-grade 35% (*v*/*v*) H_2_O_2_ (Sinopharm Chemical Reagent Co., Ltd., Beijing, China) the next day. Then, the samples were shaken thoroughly for uniform mixing, sealed in Teflon microwave digestion tubes and further digested in an accelerated microwave digestion system (Mars CEM, CEM Corporation, Matthews, NC, USA) at 800 W, 120 °C for 10 min and then 800 W, 170 °C for 30 min. The volume of each digestion was brought up to 10 mL using ultrapure water and finally filtered through 0.22 μm nylon membrane. The real samples, the certified reference material (CRM) in Tuna Fish Flesh Homogenate (IAEA-436) from the International Atomic Energy Agency (Vienna, Austria), and the procedural blanks were also treated in the same way.

Eight PTEs, namely Cd, As, Cr, Ni, Pb, Mn, Cu, and Zn, were selectively detected by using an Agilent 7500cx Inductively Coupled Plasma Mass Spectrometer (ICP-MS, Agilent Technologies, Santa Clara, CA, USA). The data for five studied PTEs (Cd, As, Cr, Ni, Pb) were expressed as µg/Kg, while the concentration for Mn, Cu, Zn were reported as mg/Kg. A quality control (QC) sample was prepared by mixing the aliquots of the real samples to represent the whole sample set and injected after every 10 real samples (to check the instrumental stability). The QC results showed <10% variation in the analytical batches. Spiked samples were also prepared in the same manner as the real meat samples, which were spiked with Cd, As, Cr, Cu, Mn, Ni, Pb and Zn at 10 and 20 µg/mL levels before the digestion. Measured concentrations were not recovery-corrected, as analytical recoveries for all elements were within the acceptable range (80–104%) and were used solely for quality control verification of method accuracy. The limits of detection (LOD) and limits of quantification (LOQ) for each PTE were calculated as three and ten times the standard deviation of procedural blanks, respectively, and are provided in Table S1.

### 2.4. Statistical Analysis

GraphPad Prism 8 was used for figures. All the statistical analysis was performed using IBM SPSS statistics (Version 27) software. Data distribution of normality were assessed using the Shapiro–Wilk test, which indicated that most variables deviated significantly from a normal distribution (*p* < 0.05). Accordingly, the data were log transformed and differences among meat types (beef, mutton, and chicken), tissue types (liver and muscle), and sampling cities were evaluated using parametric tests (including one-way ANOVA and Student’s *t*-test) and non-parametric tests (Kruskal–Wallis H test and Mann–Whitney U test). Given the exploratory nature of the study, no formal multiple-comparison correction was applied; instead, results were interpreted conservatively, emphasizing consistent patterns across tissues, species, and cities rather than individual *p*-values. This approach may increase the possibility of type I error (false positives), and therefore statistically significant associations were interpreted in conjunction with effect consistency and biological plausibility. Association of the concentrations of PTEs in the paired samples of liver and muscle were assessed by using Spearman’s rank correlation (*p* < 0.05). Multivariate pattern analysis was conducted using the MVSP (Multi-Variate Statistical Package) for PCA.

### 2.5. Average Daily Intake and Human Health Risk Assessment

The average daily intake of selected PTEs by humans was calculated using the following formula suggested by USEPA [28]:

EDI = (C × IR × EF × ED)/(BW × AT),

where EDI stands for the average daily intake (µg/kg body weight/day), C represents the PTEs’ concentration in the exposure medium (µg/kg or mg/kg), and IR is the ingestion rate (meat consumption per day). The selected ingestion rates were adopted to represent average and high-consuming population scenarios based on previously published dietary exposure and food safety studies involving meat and offal consumption [29,30,31]. Furthermore, Pakistan exhibits a relatively high per capita meat consumption (~32 kg/year) that is increasing every year, and which supports the application of conservative upper-bound intake assumptions to avoid underestimation of potential dietary exposure [20]. The upper-bound ingestion rate was applied to represent a conservative high-consumption exposure scenario among high-consuming subgroups, consistent with established food safety risk assessment practices. Therefore, the IRs were set at 20 g and 50 g of liver for low and high meat-consuming populations, respectively, whereas for muscle consumption, the IR for chicken was 250 g and 500 g and for mutton and beef, the IR was 100 g and 250 g for low and high meat-consuming populations, respectively. EF is the exposure frequency, which was 365 days/year; ED represents the exposure duration (70 years, corresponding to chronic lifetime exposure); BW is the average body weight in Pakistan (70 kg), and AT is the duration over which the dose is averaged (70 years, consistent with lifetime exposure assumptions recommended by USEPA [28] human health risk assessment guidelines). Exposure parameters, including ingestion rates, body weight, and exposure duration, were selected based on established risk assessment guidance and locally derived consumption information, and were applied as conservative screening-level assumptions for estimating non-carcinogenic and carcinogenic risks. Since elemental concentrations were determined on a dry-weight basis, regulatory comparisons and health risk estimations may represent conservative exposure scenarios, as international permissible limits are generally defined on a fresh-weight basis. This approach was adopted to avoid underestimation of potential dietary exposure.

The Human Health Risk was calculated with the help of the following equation:

THQ = EDI/RfD.

THQ stands for Target Hazard Quotient, EDI presents the Estimated Daily Intake, and RfD shows the Reference Dose suggested by USEPA [28]. A THQ > 1 was considered as a considerable risk of selected toxic elements via the consumption of chicken, mutton, and beef meat from the studied areas.

To assess cumulative non-carcinogenic health risk from simultaneous exposure to multiple PTEs, the Hazard Index (HI) was calculated as the sum of individual THQs:

HI = ∑THQi.

THQi represents the target hazard quotient of the *i*-th element. A HI > 1 indicates a potential combined health risk, whereas a HI < 1 suggests negligible risk [28]. THQ and HI calculations were based on median PTE concentrations to minimize the influence of extreme values and better represent central exposure tendencies.

## 3. Results

In this study, ninety-two paired samples of edible liver and muscle from chicken, mutton, and beef were collected from markets across four cities in Pakistan and analyzed for selected elements using ICP-MS. Overall, the concentration trend of the analyzed elements in both liver and muscle tissues followed the order: Zn > Cu > Mn > Cr > Ni > Pb > As > Cd.

### 3.1. Profiling of PTEs in the Liver and Muscle Tissues of Animals

**Figure 2:**
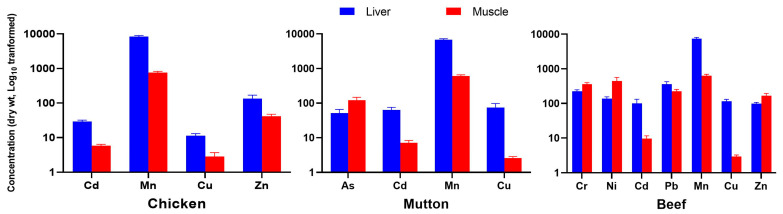
Concentrations of PTEs in the muscle and liver tissues of chicken, mutton, and beef collected from four major cities of Pakistan. Data are presented as mean ± standard error mean (SEM). Elemental concentrations are expressed as µg/kg dry weight (Log_10_ transformed), except for Zn and Cu, which are reported in mg/kg dry weight (Log_10_ transformed).

Cr concentrations (µg/kg dry weight) in beef liver (median: 205) were significantly lower (*p* < 0.05) than those in muscle samples (322.4) (Table 1, Tables S2 and S3, Figure 2). The higher Cr content in muscles may reflect its biological association with insulin action and glucose metabolism in muscles [32]. Cr concentrations in chicken and mutton did not show significant organ-wise differences (Table 1, Figure 2). Cr levels in this study were broadly consistent with values reported previously from Pakistan and other regions (Table S1). Overall, Cr concentrations in this study did not exceed the FAO/WHO permissible limit (1 µg/g), except for ~2% of beef and chicken muscle samples. Ni concentrations (µg/kg dry weight) in muscle samples ranged from 64.6 to 1159.3 (median: 224.4) for chicken, 61.8–360 (183.3) for mutton, and 124.4–1325.3 (304.2) for beef. Ni levels were significantly higher (*p* < 0.05) in beef muscle compared to beef liver (Table 1, Figure 2). No significant liver–muscle differences were observed for Ni in chicken or mutton. Observed Ni concentrations were comparable to earlier reports from Pakistan and the Middle East but differed from some African and South Asian studies (Table S1). Approximately 30% of beef and 5% of chicken samples exceeded the FAO/WHO (2011) permissible limit of 0.5 mg/kg.

**Figure 3:**
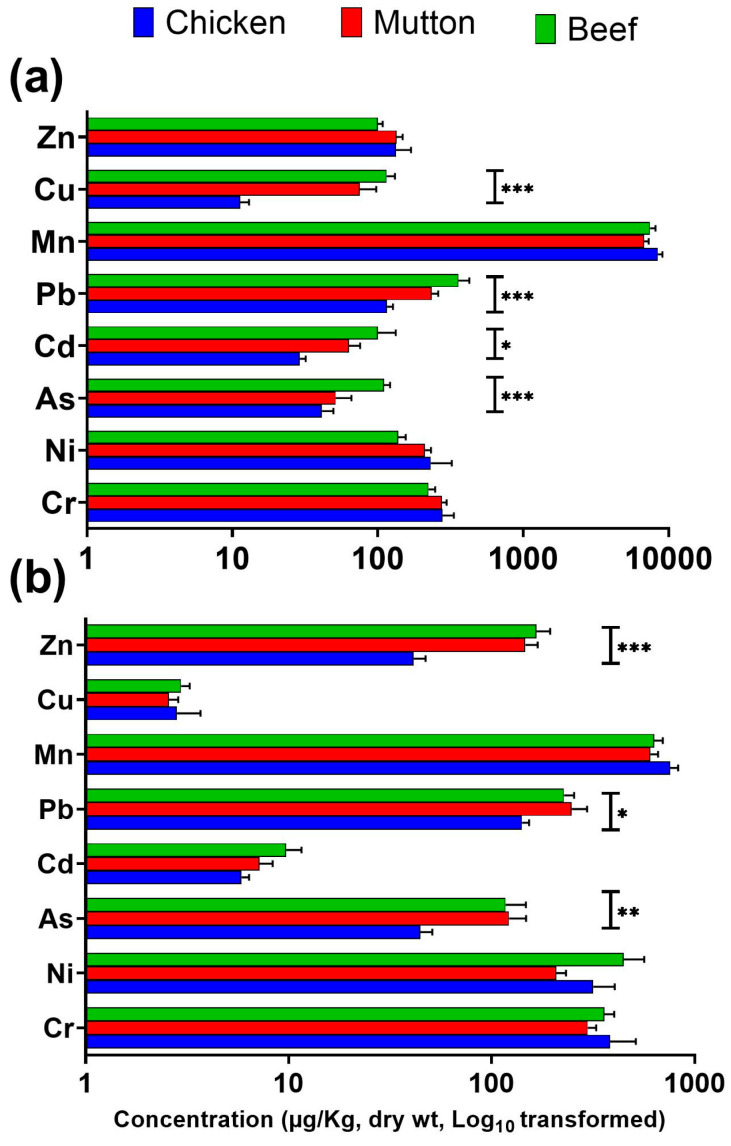
Comparison of PTE concentrations in the liver (**a**) and muscle (**b**) tissues of chicken, mutton, and beef. Bars represent mean ± SEM. Statistically significant differences among animal species are indicated by asterisks (*p* < 0.05 (*), *p* < 0.01 (**), *p* < 0.001 (***)). Elemental concentrations are expressed as µg/kg dry weight (Log_10_ transformed), except for Zn and Cu, which are reported in mg/kg dry (Log_10_ transformed) weight.

As concentrations (µg/kg dry weight) in mutton muscle (median: 83.8) were significantly higher (*p* < 0.05) than those in liver (29.1) (Table 1, Figure 2). Among species, beef liver exhibited the highest As levels (median: 106.4), showing significantly greater contamination (*p* < 0.05) compared to chicken and mutton (Table 1, Figure 3). In muscle tissues, As concentrations varied significantly and followed the descending order: mutton (28.1–271; 83.8) > beef (32.6–295.9; 63.4) > chicken (21.1–93.3; 38.9) (Table 1, Tables S2 and S3, Figure 3). All chicken samples contained As within FAO/WHO (2011) permissible limits, whereas approximately 10% of beef and mutton samples exceeded these values, indicating potential health risks to consumers. Measured As concentrations were generally higher than several regional and international reports, except those from Bangladesh (Table S1). Previous studies have suggested that chronic exposure can exceed hepatic detoxification capacity, leading to redistribution and secondary accumulation of As in muscle tissues [12,33]. Cd concentrations (µg/kg dry weight) were significantly higher (*p* < 0.05) in beef liver than in other tissues, with approximately 5% of liver samples exceeding the FAO/WHO [34] permissible limit of 0.1 mg/kg (Table 1, Figure 3). Across all species, Cd levels differed significantly between organs (*p* < 0.05), with liver consistently showing higher concentrations than muscle (Table 1, Figure 2). Overall, Cd concentrations were comparable to earlier Pakistani studies but higher than those reported from several other regions (Table S1).

Pb concentrations (µg/kg dry weight) followed a liver-dominant pattern, with significantly higher values (*p* < 0.05) in beef liver (157.8–909.6; median = 264.2) (Table 1, Figure 3). Beef muscle samples (139.3–497.2; median = 204) also contained elevated Pb levels (Figure 3). Organ-wise comparison confirmed that beef liver (median = 264.2) accumulated significantly more Pb than beef muscle (median = 204) (Table 1, Tables S2 and S3, Figure 2 and Figure 3). These values are consistent with earlier studies from Pakistan and are comparable to, or slightly higher than, levels reported in China, Africa, and the Middle East (Table S1). Approximately 10% of beef liver and muscle samples exceeded the FAO/WHO [34] limit of 0.2 mg/kg. Mn concentrations (mg/kg dry weight) were significantly higher (*p* < 0.05) in all liver samples than in the corresponding muscle tissues, with beef liver showing the highest median value (9.8 mg/kg) (Table 1, Figure 2). These concentrations were higher than those reported in studies from Pakistan and Nigeria (Table S2). Although most samples remained within the FAO/WHO limit of 1 mg/kg, a substantial proportion of liver samples and about 15% of muscle samples exceeded this threshold, indicating possible exposure through feed or environmental inputs.

Cu concentrations (mg/kg dry weight) were significantly higher (*p* < 0.05) in liver than in muscle across all animal species, with the highest median value observed in beef liver (117.2) and the lowest in chicken liver (10.0) (Table 1, Figure 2 and Figure 3). The elevated hepatic Cu levels in ruminants are attributed to limited biliary excretion capacity [35]. The current findings agree with previous reports from Pakistan (Table S1) and are comparable to those from the Middle East and Iran, while lower levels have been reported in China and Africa. Approximately 40% of liver samples exceeded the FAO/WHO [34] permissible limit of 10 mg/kg, suggesting considerable bioaccumulation potential. Zn concentrations (mg/kg dry weight) displayed clear species-specific patterns, with the highest accumulation observed in beef samples (Figure 3). In chicken, liver Zn levels (132.2) exceeded those of muscle (92.4), whereas in beef, muscle Zn concentrations (212.1) were significantly higher than liver levels (186.2; *p* = 0.037) (Table 1, Tables S2 and S3, Figure 2 and Figure 3). These results are consistent with earlier reports from Pakistan, Africa, and Europe (Table S1). The preferential Zn retention in muscle tissue may be linked to the expression of transport proteins such as ZIP4 and ZnT1 [36]. Approximately 10% of the analyzed meat samples exceeded the FAO/WHO [34] limit of 150 mg/kg, indicating localized enrichment likely related to feed supplementation practices. Overall, comparison with international datasets summarized in Table S1 indicates that the observed PTE concentrations are generally within previously reported global ranges, while selected elevated values suggest possible localized exposure influences.

### 3.2. Source Identification and Geospatial Differences in Potentially Toxic Elements

To better understand spatial and source variations in PTEs across different regions, geospatial and multivariate analyses were performed.

#### 3.2.1. City-Wise Distribution and Spatial Variations

**Figure 4:**
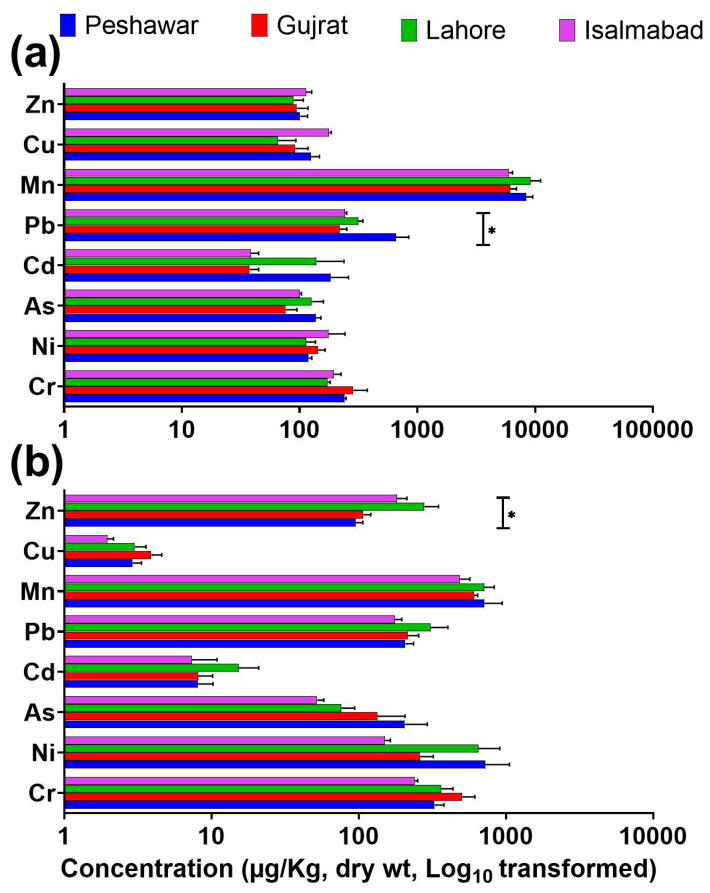
Spatial comparison of PTE concentrations in beef liver (**a**) and muscle (**b**) samples collected from Peshawar, Gujrat, Lahore, and Islamabad. Bars represent mean ± SEM. Statistically significant differences among cities are indicated by asterisks (*p* < 0.05). Elemental concentrations are expressed as µg/kg dry weight (Log_10_ transformed), except for Zn and Cu, which are reported in mg/kg dry weight (Log_10_ transformed).

Distinct spatial variations in the concentrations of PTEs were observed among beef, mutton, and chicken samples collected from major cities of Pakistan (Figure 4 and Figure S1). Chromium (Cr) exhibited non-significant higher levels (*p* < 0.05) in beef muscle samples from Gujrat compared with those from Lahore, Peshawar, and Islamabad, respectively (Figure 4 and Figure S1). Sharaf et al. [37] reported exceptionally elevated Cr levels (up to 5–8 mg/kg) in buffalo and cow organs along the industrial drain areas of Lahore, Pakistan. Cr levels in chicken samples were comparable or lower than those reported by Imran et al. [23], Iqbal et al. [38], and Abdel-Salam et al. [25] from selected regions of the Punjab and KPK provinces, indicating regional differences in feed composition and environmental contamination.

City-wise comparison further revealed that beef muscle contained non-significant higher Ni concentrations (*p* < 0.05) in samples from Lahore (median: 550 µg/kg) and Peshawar (600 µg/kg) than those from Islamabad and Gujrat (Figure 4 and Figure S1). In contrast, Pb concentrations were notably elevated (*p* < 0.05) in Peshawar compared to other cities, suggesting potential influence from vehicular emissions and industrial activities prevalent in that region (Figure 4 and Figure S1).

For Cu, beef liver samples collected from Islamabad showed non-significantly higher levels (*p* < 0.05) than those from Peshawar, Gujrat, and Lahore (Figure 4 and Figure S1). These Cu concentrations were substantially higher than those previously reported in broiler chickens from different regions of Pakistan [15,24,39]. Beef muscle samples from Lahore exhibited significantly greater Zn contents (*p* < 0.05) than those from Islamabad, Gujrat, and Peshawar (Figure 4 and Figure S1). The Zn values exceeded those documented by Arif et al. [39], Abbas et al. [15], and Khan et al. [24], indicating that local feed composition and environmental inputs may strongly influence Zn accumulation in animal tissues.

#### 3.2.2. Correlation Analysis and Source Attribution

**Figure 5:**
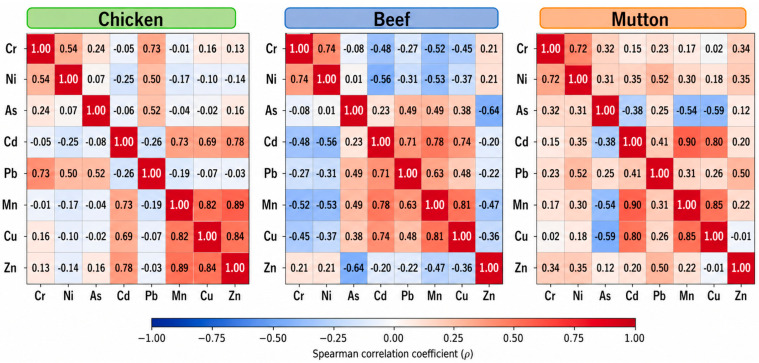
Spearman correlation matrices showing inter-element relationships among PTEs in chicken, mutton, and beef samples. Color gradients represent the strength and direction of correlation coefficients, ranging from −1 (strong negative correlation) to +1 (strong positive correlation), with numerical values displayed in each cell.

Correlation and PCA were conducted to elucidate the potential sources of metal contamination (Figure 5, Figures S2 and S3). Multiple correlation runs were performed for different animal species and tissue types to identify co-association patterns among elements.

In chicken, a strong and significant correlation (*p* < 0.01) was observed between Cr–Ni (r = 0.53), Cr–Pb (r = 0.72), As–Pb (r = 0.51), and Ni–Pb (r = 0.50). Similarly, Cd–Mn (r = 0.73), Cd–Cu (r = 0.69), Cd–Zn (r = 0.78), Mn–Cu (r = 0.82), Mn–Zn (r = 0.89), and Cu–Zn (r = 0.83) were also significant (*p* < 0.05) (Figure 5). These associations indicate shared exposure pathways and co-accumulation patterns, suggesting potential common dietary or environmental sources of exposure in poultry production systems. High Zn correlation with Cd, Cu, and Mn may reflect the role of dietary mineral supplementation, a common practice in the poultry industry to prevent deficiency-related disorders.

Previous studies from Sindh province confirmed the presence of Cu, Zn, Cd, and Pb in commercial poultry feed [40,41], indicating that contaminated feed may represent a plausible exposure route rather than a definitive source. Additionally, the proximity of poultry farms to urban areas may enhance metal uptake through airborne dust and anthropogenic emissions, further increasing PTE levels in chicken tissues.

In mutton, significant positive correlations (*p* < 0.01) were found between Cr–Ni (r = 0.72), Ni–Pb (r = 0.52), Cd–Mn (r = 0.90), Cd–Cu (r = 0.80), and Mn–Cu (r = 0.85). Weaker but still significant correlations (*p* < 0.05) were observed for Cd–Pb (r = 0.41) and Pb–Zn (r = 0.49), while As–Mn (r = −0.54) and As–Cu (r = −0.59) showed strong negative relationships (Figure 5). These trends highlight mixed sources of contamination—contaminated fodder, soil ingestion, and dust deposition during grazing—as likely contributors. 

The widespread use of agricultural chemicals such as pesticides and fertilizers has also been implicated in elevated PTE levels in the soil–plant–animal continuum [4,7]. Additionally, As bioaccumulation may result from drinking contaminated groundwater, as many regions of Pakistan are known to have high geogenic As in soil, water, and crops.

#### 3.2.3. PCA and Tissue-Specific Accumulation Patterns

Principal component analysis (PCA) was applied as an exploratory tool to identify patterns of co-variation and tissue-specific accumulation of PTEs (Figure S2). As PCA reveals statistical associations rather than direct evidence of causal source attribution, interpretations regarding potential contamination sources should be considered as indicative rather than conclusive and should be supported by targeted environmental validation. The PCA eigenvalues indicated that PC1 explained 66.06% of the total variance, followed by PC2 (11.77%) and PC3 (6.02%). Elements with high loadings on PC1 (Cr, Ni, As, Zn) were mainly associated with muscle tissues, suggesting their greater affinity for deposition in muscular systems. Conversely, PC2, dominated by Cu, Cd, Mn, and Pb, reflected their preferential accumulation in liver tissue.

These results highlight tissue-specific accumulation behavior and shared exposure pathways, consistent with the biological roles and toxicokinetics of trace metals. Metals absorbed from feed or soil enter systemic circulation and distribute organ-specifically depending on their binding affinity, metabolic demand, and detoxification mechanisms. The liver and kidneys serve as primary accumulation sites for PTEs due to their role in filtration and metal sequestration [30]. Specifically, Cd, Pb, Mn, and Cu displayed strong hepatic affinity, aligning with previous findings that liver acts as a key reservoir for these metals [29].

In the present study, Cu and Mn accumulation in liver tissues can be attributed to the organ’s central role in Cu and Mn homeostasis and storage regulation [30,42]. Excess Mn is excreted via bile, a process essential to maintain blood Mn levels and prevent neurotoxic accumulation [43]. Similarly, Cd and Pb concentrations were also significantly higher in liver than in muscle, consistent with their detoxification through hepatic metallothionein binding and biliary excretion pathways [17,44]. In contrast, As, Ni, Zn, and Cr exhibited higher affinity toward muscle tissues (Figure S2), indicating tissue-specific metabolic or structural roles. Chromium, in particular, plays a crucial physiological role as a component of the glucose-tolerance factor (GTF), which enhances insulin action and glucose metabolism [32]. The presence of Cr in muscles is therefore likely related to its involvement in insulin-mediated nutrient utilization. Moreover, dietary Cr supplementation has been reported to increase lean muscle mass and reduce fat content in livestock [45], suggesting that muscle deposition reflects both metabolic demand and dietary exposure.

Similarly, Zn and Ni enrichment in muscle can be explained by their essential enzymatic functions. Zn acts as a cofactor for numerous enzymes, such as carbonic anhydrase, and is crucial for muscle contraction, repair, and skeletal development [18,30,36]. Ni, a structural component of several metalloenzymes including urease, contributes to ruminal nitrogen metabolism and protein synthesis [42]. Hence, moderate Zn and Ni accumulation in muscle tissues represents both nutritional utilization and environmental input rather than purely toxic exposure. Bioaccumulation pf As, particularly in mutton and beef, may be influenced by chronic exposure through contaminated groundwater and forage, although definitive source attribution requires targeted environmental validation. The liver metabolizes As via the AS3MT enzyme, converting it into methylated metabolites (MMAs, DMAs) that are either stored in adipose tissue or excreted via urine and feces [12].

PC3, accounting for 6.02% of total variability, revealed contrasting elemental associations that may reflect differing environmental and anthropogenic influences. Specifically, As and Pb were potentially associated with groundwater and atmospheric dust, whereas Cr and Mn may be linked to industrial activities and feed mineralization practices.

#### 3.2.4. Geospatial PCA and Source Differentiation

The PCA conducted on city-wise data (Figure S3) provided further evidence of geographically distinct contamination pathways. High loadings of Cr, Ni, As, and Zn were observed for Lahore and Islamabad, while Cd, Mn, Ni, Cu, and Pb were more dominant in Gujrat and Peshawar. These results may suggest possible associations of Lahore and Islamabad with a combination of industrial emissions, traffic-related dust, and geogenic contamination from As- and Cr-rich aquifers [3,7]. Conversely, Gujrat and Peshawar may reflect potential associations with agricultural land use, fertilizer and pesticide application, and local feed production contributing to higher Cd, Mn, Cu, and Pb accumulation. However, these multivariate associations should not be interpreted as direct evidence of causal source attribution, but rather as indicative patterns requiring confirmation through targeted environmental sampling and source-specific validation. Overall, the integrated correlation and PCA findings highlight three dominant co-variation patterns and suggest possible contamination pathways across the studied regions: (i) geogenic groundwater influences associated with As, Cr, and Ni; (ii) atmospheric and industrial emissions linked with Pb, Cd, and Mn; (iii) mineral premix feed additives, potentially responsible for elevated Zn and Cu, particularly in poultry tissues. These inferred pathways reflect statistical associations rather than confirmed sources and should therefore be interpreted cautiously. These insights underscore the complex interplay of environmental and dietary exposure routes driving PTE accumulation in Pakistani retail meat, while acknowledging the inferential limitations of multivariate statistical approaches.

### 3.3. Estimated Daily Intake (EDI) and Associated Health Risks

Across Asia, chicken remains the most widely consumed meat, followed by beef and mutton, making these dietary sources significant pathways for human exposure to PTEs. Dietary exposure was evaluated using EDI, THQ, and HI to comprehensively assess both nutritional contribution and toxicological risk associated with metal intake. While essential trace elements such as Zn, Cu, Mn, and Cr are required for normal physiological processes, excessive intake may impair hepatic, pancreatic, and renal function, disrupt insulin regulation, and induce neurotoxic or carcinogenic effects. Conversely, non-essential elements (Ni, Pb, As, and Cd) are harmful even at low concentrations—Pb and As are established neuro- and carcinogenic agents, Ni is linked to genotoxicity and allergic reactions, and Cd is associated with renal, hepatic, and pulmonary toxicity. Therefore, comparing measured intake values against reference thresholds is crucial to determine which elements pose the greatest health risk.

**Figure 6:**
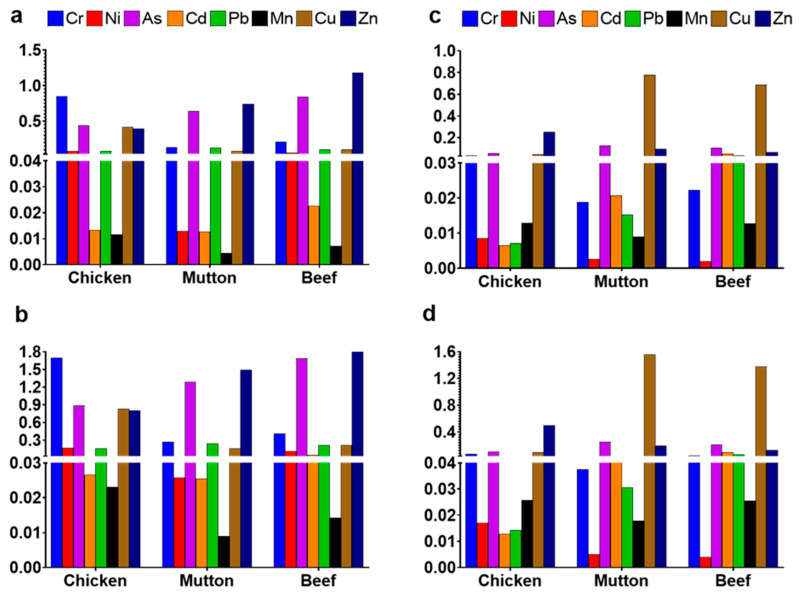
THQ values for PTEs associated with dietary exposure through consumption of chicken, mutton, and beef by (**a**) low muscle-consuming, (**b**) high muscle-consuming, (**c**) low liver-consuming, and (**d**) high liver-consuming population groups. The horizontal dashed line represents the threshold risk value (THQ = 1), above which potential non-carcinogenic health risks may occur.

Among all elements, Zn exhibited the highest intake range (19.6–710 µg/kg bw/day; Table 2), with particularly elevated levels in beef muscle (355, 710) and chicken liver (75, 151) for low and high consumers, respectively. These values are comparable to, or higher than, those reported in Pakistan [46], Africa [30,47,48], and Europe [31]. The tolerable daily intake (TDI) for Zn is 300–1000 µg/kg bw/day [49]. Current findings suggest that beef and mutton muscle are valuable Zn sources, yet a THQ > 1 in some beef samples (particularly from older animals) warrants caution (Figure 6). Cu intake ranged between 1.89 and 62.2 µg/kg bw/day (Table 2), with the highest values detected in mutton liver (31.1, 62.2) and chicken muscle (16.7, 33.4) for low and high consumption groups. These concentrations exceeded those reported in China [17] and Egypt [47] but were similar to African reports [30,48]. The WHO allowable daily intake for Cu is 40 µg/kg bw/day, and this threshold was surpassed by high liver consumers, suggesting potential health risks. THQ values > 1 for Cu in mutton and beef liver (Figure 6) reinforce this concern. Consequently, populations with high liver intake may face elevated health risks due to excessive Cu accumulation.

Mn exposure through meat consumption was comparatively low (0.63–3.59 µg/kg bw/day) across all species. The highest concentrations were observed in chicken liver (1.79, 3.5) and chicken muscle (1.62, 3.1) for low and high consumers (Table 2). The acceptable daily intake for Mn is 1.8–2.3 mg/day [46], and all current values were within this safe range. These results were lower than African data [30] but higher than Spanish reports [50]. THQ values were well below 1 (Figure 6), indicating negligible health risk from Mn exposure via meat. Cr contributed moderately to overall metal intake (0.056–5.1 µg/kg bw/day). Liver concentrations were highest in chicken (0.1, 0.22), followed by beef (0.06, 0.13) and mutton (0.056, 0.11), while muscle Cr levels peaked in chicken (2.54, 5.1), followed by beef (0.624, 1.24) and mutton (0.41, 0.82) (Table 2). These findings exceeded Iranian data [51] but were below those reported in China [17] and Africa [30,47]. THQ values for chicken muscle exceeded 1 (Figure 6), suggesting that frequent consumption could contribute significantly to Cr exposure.

Ni exposure ranged between 0.04 and 3.31 µg/kg bw/day (Table 2). Liver values were highest in chicken (0.17, 0.31), followed by mutton (0.051, 0.11) and beef (0.04, 0.07), while muscle values followed a similar pattern: chicken (1.65, 3.31) > beef (1.13, 2.72) > mutton (0.25, 0.52). These results were higher than those from Africa [30] and Iran [51] but comparable to China [17]. All THQ values were below 1, yet given Ni’s classification as a carcinogen and sensitizer by IARC, continuous monitoring of dietary exposure is advisable (Figure 6). Human Pb intake through meat consumption ranged from 0.029 to 0.98 µg/kg bw/day (Table 2). Beef liver (0.13, 0.27) showed the highest intake among liver consumers, while mutton muscle (0.49, 0.98) was dominant among muscle samples. Present results were lower than African studies [30,47] but comparable to Chinese data [17]. Although THQ values remained below 1, Pb’s cumulative neurotoxic and carcinogenic effects imply that even trace exposures can cause adverse outcomes, especially in children and vulnerable adults (Figure 6).

Arsenic intake varied between 0.016 and 0.507 µg/kg bw/day (Table 2). The highest muscle concentrations were recorded in mutton (0.50, 0.40), and liver values were greatest in beef (0.25, 0.51). These values were higher than those reported in Africa [30] and comparable to Chinese data [17]. Although intake levels were below the 3 µg/kg bw/day limit, As remains a potent carcinogen. THQ values exceeded 1 for total As in beef and mutton muscle among high-consuming populations, indicating possible health risks (Figure 6). Once absorbed through the gastrointestinal tract, As enters the bloodstream via aquaglyceroporin channels and is directed to the liver for detoxification [33]. It should be noted that this study quantified only total arsenic without distinguishing between inorganic and organic species. Since arsenic-related carcinogenic risk is primarily driven by inorganic forms, the present health risk estimates based on total arsenic should be interpreted cautiously, and future studies should prioritize arsenic speciation analysis for refined risk assessment.

Cd concentrations ranged between 0.006 and 0.096 µg/kg bw/day (Table 2). Liver values were highest in beef (0.05, 0.10), followed by mutton (0.02, 0.04) and chicken (0.01, 0.01), while muscle levels followed a similar pattern (beef > mutton > chicken), with maxima of 0.05, 0.02, and 0.024 µg/kg bw/day, respectively. These findings are comparable or slightly higher than reports from Africa [30], Egypt [47], and China [17]. Although THQ values were below 1, the chronic nephrotoxicity of Cd warrants attention, as prolonged intake may lead to kidney dysfunction and oxidative stress (Figure 6).

To assess the cumulative non-carcinogenic health risk associated with simultaneous exposure to multiple PTEs, the HI was calculated as the sum of individual THQs. The results demonstrated pronounced variation across meat types, tissues, and consumption scenarios (Table S4). Notably, HI values exceeded the safety threshold of 1 for all high-consumption scenarios, indicating potential combined health risks. The highest cumulative risk was observed among high consumers of beef muscle (HI ≈ 3.18), followed by mutton muscle (HI ≈ 3.00) and beef liver (HI ≈ 2.26). In contrast, low liver-consuming populations exhibited HI values below unity, reflecting comparatively lower cumulative exposure. Elevated HI values were primarily driven by the combined contributions of Cu, Zn, As, and Cr, highlighting the importance of evaluating additive toxicological effects rather than relying solely on single-element risk metrics. These findings emphasize that the frequent consumption of beef and mutton, particularly muscle and liver tissues, may pose appreciable long-term health risks, underscoring the necessity for integrated dietary risk assessment frameworks and targeted mitigation strategies.

Overall, beef and mutton muscle samples exhibited the greatest accumulation of Zn, As, and other PTEs, representing potential chronic exposure sources. Chicken muscle was generally safe, except for Cr, whereas chicken liver displayed sporadically high contamination and should be consumed sparingly. Mutton (liver and muscle) and chicken muscle remain valuable dietary sources of essential nutrients such as Zn. However, cumulative THQ analyses indicated that beef (liver and muscle) and chicken liver pose higher combined risks. Although some samples met WHO guideline values, emerging evidence suggests current limits may not fully protect against chronic exposure effects. Therefore, even minimal concentrations of Cd, Pb, and As require careful monitoring, especially in environmentally impacted regions. The elevated intake and THQ values for Cr, As, Zn (in muscle), and Cu (in liver) observed in this study emphasize the need for comprehensive mitigation strategies to minimize PTE contamination in meat and reduce human exposure. Recommended interventions include safe feed practices, water treatment for livestock, judicious fertilizer and pesticide use, improved livestock management, adoption of cleaner industrial technologies, regular soil and water monitoring, and public awareness programs on safe meat consumption and chemical exposure risks.

### 3.4. Study Limitations and Uncertainty Considerations

Several limitations of this study should be acknowledged. Reporting elemental concentrations on a dry-weight basis may yield conservative estimates of regulatory exceedance and dietary exposure when compared with fresh-weight-based guideline values. Although this approach minimizes analytical variability associated with moisture content and enhances inter-study comparability, it may modestly overestimate real-world exposure. Furthermore, dietary exposure and health risk assessments inherently involve uncertainties associated with inter-individual consumption variability, regional dietary habits, food preparation practices and cooking effects (e.g., losses or concentration changes during processing), bioavailability, lifetime exposure assumptions, and parameter choices (e.g., ingestion rates and body weight). These uncertainties may influence THQ and HI estimates. Accordingly, the calculated EDI, THQ, and HI values should be interpreted as conservative upper-bound estimates intended to support precautionary public health evaluation rather than precise individual exposure predictions. In addition, arsenic was quantified only as total concentration in this study; however, toxicity and carcinogenic risk are primarily driven by inorganic arsenic species. Therefore, arsenic-related health risk estimates should be interpreted cautiously, as use of total arsenic may overestimate risk where less toxic organic arsenic species contribute to measured concentrations. Future studies should prioritize arsenic speciation analysis and parallel reporting of fresh-weight concentrations to refine exposure characterization and improve regulatory relevance. Where moisture content conversion data are available, paired dry-weight and fresh-weight concentrations should also be reported to improve comparability with FAO/WHO limits and other food safety benchmarks.

Moreover, detailed information on animal-related variables (e.g., age, sex, breed), as well as feed composition, farming practices (organic versus conventional systems), and drinking water sources was not consistently available from retail supply chains. The absence of these variables may contribute to variability in elemental accumulation patterns and influence interpretation of species- and tissue-specific differences. Because sampling was market-based rather than fully randomized, some selection bias cannot be excluded, and future studies using stratified random sampling would improve representativeness. Finally, the cross-sectional design provides a snapshot of contamination levels and does not capture seasonal or temporal variability, highlighting the need for longitudinal and multi-seasonal monitoring to better characterize exposure dynamics.

## 4. Conclusions

This study provides essential baseline data on potentially toxic elements (PTEs) in the muscle and liver tissues of chicken, mutton, and beef collected from four major cities of Pakistan, using ICP-MS analysis. Among the studied samples, beef muscle exhibited the highest PTE bioaccumulation, with a THQ > 1 for As, Cr, Cu, and Zn, indicating potential chronic health risks. Mutton muscle also showed elevated levels of Zn and As, while chicken muscle appeared generally safe except for Cr. In contrast, chicken liver displayed higher contamination and should therefore be consumed in moderation. Despite these concerns, mutton (liver and muscle) and chicken muscle remain good dietary sources of essential macronutrients such as Zn. Up to 40% of beef liver and 10% of muscle samples exceeded FAO/WHO permissible limits for at least one PTE. Multivariate analysis identified strong co-variation patterns, suggesting potential associations with groundwater inputs, atmospheric dust deposition, and feed supplementation practices; however, definitive source attribution requires further targeted environmental investigations. The study is cross-sectional in nature, restricted to four urban supply zones, and does not account for seasonal variability, animal age, feed composition, or cooking effects, which should be addressed in future investigations. Although conducted on a limited scale, the findings emphasize an urgent need for improved feed and water quality monitoring, sustainable livestock management practices, and revised national consumption guidelines to mitigate dietary exposure and safeguard public health.

## Figures and Tables

**Table 1:** Descriptive statistics of potentially toxic element (PTE) concentrations (µg/kg, dry weight) in the liver and muscle tissues of chicken, mutton, and beef collected from retail markets in Pakistan. Data are presented as mean ± SD, median, and range (minimum–maximum). Statistical differences among animal species within each tissue type were evaluated using the Kruskal–Wallis test; *p* < 0.05 and **bold** values indicate statistically significant differences. The concentration for Mn, Cu, Zn are as mg/Kg.

**Liver**									
Animal	Stat parameter	Cr	Ni	As	Cd	Pb	Mn	Cu	Zn
Chicken	Mean ± STD	278.7 ± 187.3	231.3 ± 315.1	41.3 ± 28.3	29.1 ± 9.8	115.5 ± 39.9	8.4 ± 2.1	11.3 ± 5.8	132.9 ± 124.7
	Median	220.6	111.3	31.8	24.5	102.1	8.4	10.0	103.8
	Min–Max	113.2–705.4	64.4–1193.0	19.0–112.4	18.8–45.4	83.0–200.9	4.7–12.6	3.0–26.1	67.5–525.3
Mutton	Mean ± STD	275.6 ± 74.6	211.0 ± 71.9	51.3 ± 50.6	63.3 ± 42.6	234.9 ± 87.0	6.8 ± 1.7	74.5 ± 78.0	134.6 ± 46.4
	Median	260.5	203.5	29.1	53.3	218.9	7.1	42.1	138.3
	Min–Max	167.2–395.0	100.9–356.2	18.0–164.4	19.1–145.1	116.2–428.2	3.0–8.8	4.7–218.0	50.7–200.9
Beef	Mean ± STD	223.5 ± 84.1	138.2 ± 60.1	110.2 ± 38.7	100.0 ± 113.7	358.6 ± 233.2	7.4 ± 2.3	115.1 ± 55.2	99.6 ± 28.4
	Median	205.0	131.7	106.4	44.0	264.2	6.8	117.2	107.6
	Min–Max	143.2–466.6	52.2–278.2	40.7–171.9	23.6–336.8	157.8–909.6	5.1–12.5	11.9–192.8	48.6–137.5
*p* ˂ 0.05 is significant		0.135	0.062	**0.001**	**0.018**	**0.000**	0.201	**0.000**	0.100
**Muscle**									
Animal		Cr	Ni	As	Cd	Pb	Mn	Cu	Zn
Chicken	Mean ± STD	383.2 ± 450.0	316.9 ± 299.8	44.5 ± 22.5	5.9 ± 1.8	141.1 ± 40.5	0.8 ± 0.3	2.8 ± 3.0	41.3 ± 20.4
	Median	233.6	224.4	38.9	5.8	129.3	0.7	1.9	36.5
	Min–Max	152.2–1781.9	64.6–1159.3	21.1–93.3	3.6–9.3	97.9–225.9	0.5–1.1	0.7–11.7	20.4–83.9
Mutton	Mean ±	297.3 ± 103.4	208.2 ± 82.9	121.6 ± 90.1	7.2 ± 4.0	247.7 ± 164.1	0.6 ± 0.2	2.6 ± 1.0	146.6 ± 75.5
	Median	273.2	183.8	83.0	6.2	185.3	0.6	2.5	136.0
	Min–Max	164.4	61.8	28.1	1.4	123.1	0.3	1.1	51.5
Beef	Mean ± STD	359.3 ± 145.0	446.7 ± 406.3	117.2 ± 104.6	9.7 ± 6.4	227.2 ± 95.3	0.6 ± 0.2	3.0 ± 1.0	166.4 ± 95.7
	Median	322.4	304.2	63.4	7.7	204.0	0.6	2.9	126.6
	Min–Max	228.9–727.5	124.4–1325.3	32.6–295.9	2.8–26.4	139.3–497.2	0.4–12	1.7–4.9	75.9–414.2
*p* ˂ 0.05 is significant		0.245	0.256	**0.023**	0.211	**0.005**	0.369	0.234	**0.000**

**Table 2:** EDI (µg/kg body weight/day) of PTEs associated with dietary exposure through the consumption of liver and muscle tissues of chicken, mutton, and beef by low- and high-meat-consuming population groups.

EDI (µg/kg Body Weight/Day)									
Type of Sample	Low-Meat-Consuming Population								
	Animal	Cr	Ni	As	Cd	Pb	Mn	Cu	Zn
Liver	Chicken	0.101	0.170	0.016	0.006	0.029	1.79	1.89	75.04
	Mutton	0.056	0.051	0.037	0.021	0.061	1.25	31.13	28.7
	Beef	0.067	0.040	0.031	0.048	0.13	1.78	27.54	19.64
Muscle	Chicken	2.54	1.656	0.133	0.013	0.32	1.62	16.71	119.8
	Mutton	0.41	0.258	0.194	0.013	0.49	0.63	3.168	223.7
	Beef	0.62	1.136	0.254	0.023	0.42	1.004	4.23	355
	High-meat-consuming population								
Liver	Chicken	0.202	0.341	0.032	0.013	0.057	3.59	3.79	150
	Mutton	0.11	0.102	0.075	0.041	0.12	2.5	62.27	57.4
	Beef	0.133	0.079	0.063	0.096	0.26	3.56	55.1	39.29
Muscle	Chicken	5.09	3.31	0.267	0.027	0.64	3.24	33.42	239.6
	Mutton	0.82	0.51	0.387	0.025	0.98	1.26	6.33	447
	Beef	1.24	2.27	0.507	0.045	0.85	2.1	8.46	710

## Data Availability

The original contributions presented in this study are included in the article/Supplementary Material. Further inquiries can be directed to the corresponding authors.

## Supplementary Materials

The following supporting information can be downloaded at: https://www.mdpi.com/article/10.3390/jox16030090/s1, Table S1: Comparison of PTEs levels measured in present and worldwide reported data; Table S2: Concentrations (µg/kg, dry weight) of PTEs in Liver of chicken, mutton, and beef among different cities; Table S3: Concentrations (µg/kg, dry weight) of PTEs in muscle of chicken, mutton, and beef among different cities; Table S4: Cumulative Hazard Index (HI) values for PTEs associated with dietary exposure through consumption of chicken, mutton, and beef under different consumption scenarios. HI > 1 indicates potential non-carcinogenic health risk; Figure S1: Concentration (µg/kg, dry weight) of PTE in (a) Muscle and (b) Liver tissues within meat in different cities of Pakistan; Figure S2: PCA analysis for studied PTEs measured on liver and muscle of different animals; Figure S3: PCA analysis for studied PTEs measured in the studied different animals at different cities of Pakistan. References [15,17,23,24,52,53,54,55,56,57,58,59,60,61,62,63,64] are cited in the Supplementary Materials.
