# Supplementary material for: Tissue-Specific Accumulation and Dietary Risk of Arsenic and Other Potentially Toxic Elements in Retail Meats
Source: J Xenobiot. 2026 May 21;16(3):90. doi: 10.3390/jox16030090 (PMC13214749; doi:10.3390/jox16030090)
Supplement: Supplementary file 1 [file jox-16-00090-s001.zip › jox-4262199-supplementary.pdf]

Supplementary Materials: Tissue-Specific Accumulation and Dietary Risk of Arsenic and Other Potentially Toxic Elements in Retail Meats

Syed Sayyam Abbas, Syed Ali Musstjab Akber Shah Eqani, Ismat Nawaz, Mansoor A. Alghamdi, Ahmed S. Summan, Abdul Qadir, Shabbar Abbas, Iqra Rasheed, Syeda Maria Ali, Mustafa Nawaz Shafqat , Mohammed I. Orif, Heqing Shen and Nadeem Ali

List of Tables:

Table S1: Comparison of selected PTEs levels (mg/kg) measured in present and worldwide reported data.

Table S2: Concentrations (µg/kg, dry weight) of selected PTEs in Liver of chicken, mutton, and beef among different cities

Table S3: Concentrations (µg/kg, dry weight) of PTEs in muscle of chicken, mutton, and beef among different cities

Table S4. Cumulative Hazard Index (HI) values for PTEs associated with dietary exposure through consumption of chicken, mutton, and beef under different consumption scenarios. HI > 1 indicates potential non-carcinogenic health risk

List of Figures:

Figure S1 Concentration (µg/kg, dry weight) of PTEs in (a) Muscle and (b) Liver tissues within meat in different cities of Pakistan.

Figure S2 PCA analysis for studied PTEs measured on liver and muscle of different animals.

Figure S3 PCA analysis for studied PTEs measured in the studied different animals at different cities of Pakistan.

Table S1: Comparison of PTEs levels measured in present and worldwide reported data.

	Cr		Mn		Ni		Cu		Zn		As		Cd		Pb		Reference
Permissible limits	4.0 mg/kg		1 mg/kg		0.5 mg/kg		10 mg/kg		50 mg/kg		0.1 mg/kg		0.5 mg/kg		0.005 mg/kg		
LOD	0.005 mg/kg dw		0.02 mg/kg dw		0.005 mg/kg dw		0.02 mg/kg dw		0.05 mg/kg dw		0.001 mg/kg dw		0.0005 mg/kg dw		0.001 mg/kg dw		
Location	liver	muscle	Liver	muscle	liver	muscle	liver	muscle	liver	muscle	liver	muscle	liver	muscle	liver	muscle	
**Chicken (mg/kg)**																	
Pakistan	0.28	0.38	8.4	0.8	0.2	0.3	11.3	2.8	132.9	41.3	0.04	0.04	0.03	0.006	0.12	0.14	present study
Pakistan	0.1	0.06	0.5	0.4					110.2	107.4			0.7	0.6	0.3	0.2	[1]
Pakistan						0.39								0.017		0.2	[2]
China		0.06				0.042		0.54				0.018		0.003		0.06	[3]
Egypt														0.1		0.3	[4]
Iraq		0.1		0.0		0.0		0.2		2.0				0.1		1.2	[5]
Pakistan	0.8	0.8	0.4	0.4	3.9	2.8	2.8	2.4	31.9	30.9	0.3	0.6	0.5	0.4	2.3	2.4	[6]
Nigeria			4.1		1.1		1.4		3.1		0.0		0.3		0.2		[7]
Thailand		0.0														0.0	[8]
**Mutton (mg/kg)**																	
Pakistan	0.28	0.28	6.80	0.60	0.21	0.21	74.50	2.60	134.6	146.6	0.005	0.121	0.06	0.007	0.23	0.05	present study
China		0.05				0.06		1.0				0.008		0.002		0.06	[3]
Nigeria							2.0		4.0				0.0		0.2		[9]
Zambia	14.9				23.2		159.0		63.9		0.0		0.1		0.1		[10]
Slovak Republic					0.2	0.4	84.1	6.3	79.9	81.2			0.5	0.1	1.1	0.7	[11]
Ghana		1.0										0.0		0.0			[12]
Nigeria	0.8		2.8		0.1		0.5		2.3		0.3		0.8		0.2		[7]
Brazil												156.0		1281.0		660.0	[13]

**Beef (mg/kg)**																	
Pakistan	0.22	0.36	7.40	0.60	0.14	0.45	115.10	3.00	99.60	166.40	0.11	0.12	0.10	0.009	0.36	0.23	Present study
Egypt														0.1		0.2	[4]
China		0.06				0.06		0.67				0.018		0.002		0.06	[3]
Romania								1.0		42.1				0.1		0.6	[14]
Iraq		0.2		0.0		0.0		0.1		2.4				0.1		0.5	[5]
Pakistan														0.1		2.0	[15]
Ghana		0.3										0.0		0.1			[12]
Egypt	21.9	11.2			14.6	21.2					4.6	5.1	14.2	1.4	42.7	8.8	[16]
Nigeria	0.4		1.3		0.3		0.9		4.2		0.1		0.2		0.3		[7]
Thailand		0.0												0.0		0.0	[8]
North Spain															30.7	2.0	[17]

Table S2: Concentrations (μg/kg, dry weight) of PTEs in Liver of chicken, mutton, and beef among different cities

		Cr	Ni	As	Cd	Pb	Mn(mg/kg)	Cu(mg/kg)	Zn(mg/kg)
**Peshawar**									
Chicken	Mean ± STD	329.2 ± 239.27	141.73 ± 73.62	54.14 ± 50.66	35.84 ± 9.81	129.95 ± 62.51	7.52 ± 0.81	11.89 ± 2.2	238.91 ± 248.23
	Median	239.49	149.83	29.39	38.55	105.96	7.3	13.01	105.52
	Min - Max	147.75 - 600.36	64.41 - 210.97	20.62 - 112.42	24.97 - 44.01	82.99 - 200.9	6.85 - 8.42	9.35 - 13.3	85.88 - 525.32
Mutton	Mean ± STD	254.92 ± 34.97	154.77 ± 21.44	27.63 ± 6.24	30.1 ± 13.63	152.62 ± 34.73	6.34 ± 0.7	22.38 ± 10.25	109.15 ± 21.37
	Median	261	161.79	26.3	25.91	156.18	5.97	19.58	100.59
	Min - Max	217.31 - 286.45	130.7 - 171.82	22.16 - 34.43	19.05 - 45.32	116.24 - 185.43	5.89 - 7.15	13.82 - 33.74	93.39 - 133.47
Beef	Mean ± STD	241.07 ± 11.81	118.98 ± 13.88	137.41 ± 24.38	184.52 ± 130.3	657.62 ± 326.76	8.44 ± 1.92	124.74 ± 38.97	101.23 ± 27.03
	Median	238.34	113.25	130.47	241.78	774.88	7.76	132.61	108.29

	Min - Max	230.88 - 254.01	108.87 - 134.81	117.26 - 164.51	35.39 - 276.38	288.41 - 909.57	6.95 - 10.6	82.44 - 159.18	71.36 - 124.02
**Gujrat**									
Chicken	Mean ± STD	194.02 ± 47.26	96.33 ± 16.8	32.47 ± 3.3	20.57 ± 2.52	103.02 ± 17.68	8.86 ± 3.24	10.47 ± 2.16	97.39 ± 18.69
	Median	210.24	94.58	34.14	19.45	101.32	7.25	10.6	87.42
	Min - Max	140.79 - 231.03	80.47 - 113.94	28.67 - 34.61	18.81 - 23.45	86.25 - 121.49	6.74 - 12.58	8.25 - 12.55	85.79 - 118.95
Mutton	Mean ± STD	318.2 ± 130.81	250.77 ± 133.3	109.37 ± 78.17	61.68 ± 15.44	315.49 ± 137.48	6.2 ± 2.92	97.63 ± 103.78	142.33 ± 80.38
	Median	392.49	295.13	143.86	69.87	355.94	6.99	78.53	175.39
	Min - Max	167.16 - 394.96	100.94 - 356.24	19.89 - 164.38	43.87 - 71.3	162.32 - 428.21	2.97 - 8.65	4.73 - 209.65	50.69 - 200.9
Beef	Mean ± STD	283.62 ± 159.05	143.53 ± 36.14	76.08 ± 32.93	37.61 ± 12.52	219.08 ± 56.77	6.16 ± 1.35	91.46 ± 46.11	94.34 ± 40.95
	Median	205.43	142.19	81.75	41.74	229.46	5.62	71.76	106.82
	Min - Max	178.8 - 466.63	108.07 - 180.32	40.69 - 105.8	23.55 - 47.55	157.83 - 269.94	5.16 - 7.69	58.47 - 144.14	48.6 - 127.6
**Lahore**									
Chicken	Mean ± STD	336.05 ± 322.14	554.11 ± 567.48	27.49 ± 14.52	25.83 ± 5.29	101.97 ± 22.58	9.15 ± 0.84	13.73 ± 10.75	83.48 ± 17.8
	Median	189.57	360.59	19.26	24.01	93.2	9.06	7.69	80.31
	Min - Max	113.17 - 705.39	108.71 - 1193.04	18.95 - 44.26	21.69 - 31.79	85.09 - 127.62	8.36 - 10.03	7.35 - 26.14	67.47 - 102.65
Mutton	Mean ± STD	279.85 ± 70.33	239.97 ± 29.66	26.73 ± 7.71	83.12 ± 57.85	236.52 ± 30.89	7.68 ± 1.2	92.88 ± 110.18	159.23 ± 34.06
	Median	252.96	232.64	29.47	95.42	223.73	7.91	50.53	148.62

	Min - Max	226.94 - 359.65	214.67 - 272.62	18.02 - 32.69	20.11 - 133.82	214.07 - 271.75	6.39 - 8.76	10.15 - 217.95	131.74 - 197.34
Beef	Mean ± STD	173.03 ± 15.07	113.73 ± 38.75	126.9 ± 55.79	139.4 ± 171.03	314.59 ± 52.3	9.12 ± 3.48	65.58 ± 47.43	89.18 ± 31.96
	Median	166.08	128.59	144.39	46.28	334.24	9.38	82.94	82.42
	Min - Max	162.7 - 190.33	69.75 - 142.85	64.46 - 171.86	35.15 - 336.78	255.31 - 354.22	5.52 - 12.46	11.91 - 101.89	61.15 - 123.98
					**Islamabad**				
Chicken	Mean ± STD	255.48 ± 96.94	132.89 ± 90.52	51.18 ± 28.64	34.18 ± 12.85	127.06 ± 54.89	8.15 ± 3.29	9.24 ± 6.7	111.78 ± 11.46
	Median	272.54	97.71	40.33	36.96	102.96	8.63	8.47	105.47
	Min - Max	151.14 - 342.75	65.24 - 235.72	29.55 - 83.66	20.16 - 45.4	88.34 - 189.88	4.66 - 11.18	2.96 - 16.3	104.86 - 125
Mutton	Mean ± STD	249.52 ± 53.78	198.29 ± 26.95	41.5 ± 30	78.25 ± 60.22	235.05 ± 26.48	6.9 ± 2.03	87.02 ± 69.59	127.5 ± 41.61
	Median	260	192.41	28.67	61.35	244.18	7.52	107.42	143.1
	Min - Max	191.27 - 297.28	174.76 - 227.7	20.06 - 75.79	28.28 - 145.11	205.21 - 255.76	4.64 - 8.55	9.5 - 144.13	80.34 - 159.06
Beef	Mean ± STD	196.13 ± 49.23	176.45 ± 114.7	100.44 ± 5.75	38.51 ± 10.88	243.12 ± 14.43	5.98 ± 0.77	178.79 ± 12.19	113.83 ± 22.43
	Median	204.63	198.93	97.45	34.22	240.92	6.21	172.72	111.05
	Min - Max	143.2 - 240.55	52.16 - 278.24	96.82 - 107.07	30.44 - 50.89	229.91 - 258.52	5.12 - 6.62	170.83 - 192.83	92.92 - 137.52

Table S3: Concentrations (μg/kg, dry weight) of PTEs in muscle of chicken, mutton, and beef among different cities

		Cr	Ni	As	Cd	Pb	Mn(mg/kg)	Cu(mg/kg)	Zn (mg/kg)
					**Peshawar**				
Chicken	Mean ± STD	267.71 ± 91.99	216.16 ± 116.55	45.1 ± 14.53	7.51 ± 1.69	151.61 ± 64.72	0.68 ± 0.29	2.53 ± 1.11	35.26 ± 16.19
	Median	245.18	181.38	40.29	7.3	121.04	0.53	2.43	31.6
	Min - Max	189.08 - 368.87	120.96 - 346.14	33.58 - 61.42	5.93 - 9.29	107.83 - 225.95	0.49 - 1.01	1.47 - 3.68	21.21 - 52.97
Mutton	Mean ± STD	231.33 ± 58.09	170.63 ± 99.35	165.5 ± 124.62	5.08 ± 3.26	176.12 ± 49.19	0.57 ± 0.1	2.6 ± 0.41	121.85 ± 53.15
	Median	260.55	193.55	197.15	6.4	182.77	0.52	2.43	104.72
	Min - Max	164.43 - 269	61.82 - 256.52	28.1 - 271.24	1.37 - 7.48	123.94 - 221.64	0.51 - 0.69	2.31 - 3.07	79.38 - 181.46
Beef	Mean ± STD	327.95 ± 90.25	724.91 ± 576.23	205.73 ± 149.97	8.08 ± 3.74	207.91 ± 48.44	0.72 ± 0.4	2.92 ± 0.74	95.36 ± 20.24
	Median	328.81	673.09	288.72	6.08	192.5	0.52	3.2	93.93
	Min - Max	237.28 - 417.77	176.33 - 1325.29	32.61 - 295.86	5.76 - 12.4	169.06 - 262.18	0.46 - 1.17	2.08 - 3.48	75.87 - 116.29
					**Gujrat**				
Chicken	Mean ± STD	217.73 ± 87.79	120.35 ± 71.44	37.78 ± 27.73	6.22 ± 2.31	117.9 ± 27.63	0.7 ± 0.22	1.34 ± 0.33	31.12 ± 9.27
	Median	182.67	95.54	22.42	7.47	106.39	0.68	1.51	36.23
	Min - Max	152.89 - 317.63	64.61 - 200.88	21.13 - 69.78	3.56 - 7.65	97.88 - 149.43	0.48 - 0.93	0.96 - 1.55	20.41 - 36.7
Mutton	Mean ± STD	258.97 ± 20.28	155.15 ± 1.46	164.58 ± 115.21	7.31 ± 2.91	340.27 ± 304.88	0.66 ± 0.17	1.81 ± 0.78	135 ± 36.11
	Median	253.26	155.26	195.78	6.01	185.38	0.65	1.69	149.47
	Min - Max	242.15 - 281.49	153.63 - 156.55	36.98 - 260.99	5.28 - 10.64	143.92 - 691.5	0.5 - 0.83	1.09 - 2.65	93.91 - 161.63

Beef	Mean ± STD	502.77 ± 195.54	260.56 ± 104.56	134.43 ± 124.97	8.16 ± 3.54	215.99 ± 68.84	0.61 ± 0.06	3.91 ± 1.2	107.86 ± 22.78
	Median	409.46	245.07	65.2	7.48	236.12	0.59	4.21	115.67
	Min - Max	371.37 - 727.49	164.61 - 372.01	59.41 - 278.69	5.01 - 11.99	139.33 - 272.53	0.56 - 0.68	2.59 - 4.94	82.2 - 125.72
**Lahore**									
Chicken	Mean ± STD	257.23 ± 80.03	378.27 ± 121.1	60.61 ± 32.61	4.73 ± 1.3	135.77 ± 38.32	0.87 ± 0.35	2.43 ± 1.68	56.44 ± 32.09
	Median	222	399.78	60.36	4.07	134.82	1.01	2.5	64.29
	Min - Max	200.85 - 348.83	247.87 - 487.18	28.12 - 93.34	3.88 - 6.23	97.93 - 174.55	0.47 - 1.13	0.71 - 4.07	21.15 - 83.86
Mutton	Mean ± STD	373.23 ± 180.95	258.08 ± 94.74	106.86 ± 44.46	9.45 ± 7.27	232.42 ± 136.26	0.51 ± 0.33	2.52 ± 1.67	87.92 ± 35.53
	Median	321.57	239.5	99.44	5.44	189.09	0.34	1.68	89.82
	Min - Max	223.73 - 574.39	174 - 360.73	66.58 - 154.57	5.07 - 17.85	123.09 - 385.07	0.3 - 0.88	1.43 - 4.44	51.48 - 122.46
Beef	Mean ± STD	365.5 ± 124.28	650.34 ± 450.42	76.29 ± 30.85	15.33 ± 9.75	308.21 ± 165.04	0.71 ± 0.21	3.02 ± 1.01	280.17 ± 119.13
	Median	316.07	418.2	80.83	11.6	235.1	0.61	3.23	239.95
	Min - Max	273.55 - 506.89	363.34 - 1169.48	43.42 - 104.61	8 - 26.4	192.35 - 497.18	0.58 - 0.95	1.92 - 3.9	186.36 - 414.2
**Islamabad**									
Chicken	Mean ± STD	790.07 ± 870.6	552.76 ± 546.59	34.38 ± 11.54	5.02 ± 0.6	159.28 ± 33.23	0.78 ± 0.23	4.99 ± 5.85	42.6 ± 18.81
	Median	436.04	400.54	37.53	5.02	164.55	0.74	2.29	48.08
	Min - Max	152.25 - 1781.91	98.43 - 1159.33	21.59 - 44.02	4.42 - 5.61	123.73 - 189.56	0.57 - 1.02	0.97 - 11.7	21.66 - 58.06
Mutton	Mean ± STD	325.66 ± 61.03	249.13 ± 79.53	49.61 ± 12.19	6.88 ± 1.53	241.98 ± 115.29	0.68 ± 0.15	3.36 ± 0.26	241.72 ± 78.02
	Median	305.41	263.75	49.62	6.47	185.17	0.72	3.33	253.46

	Min - Max	277.33 - 394.25	163.29 - 320.33	37.42 - 61.79	5.59 - 8.58	166.11 - 374.65	0.52 - 0.81	3.11 - 3.63	158.49 - 313.2
Beef	Mean ± STD	240.94 ± 18.69	150.92 ± 23.01	52.34 ± 9.72	7.39 ± 6.12	176.49 ± 34.79	0.49 ± 0.14	1.97 ± 0.34	182.34 ± 52.55
	Median	231.4	162.99	53.37	4.99	165.32	0.43	1.8	187.5
	Min - Max	228.94 - 262.48	124.39 - 165.39	42.14 - 61.51	2.83 - 14.34	148.65 - 215.49	0.38 - 0.65	1.74 - 2.36	127.4 - 232.11

Table S4. Cumulative Hazard Index (HI) values for PTEs associated with dietary exposure through consumption of chicken, mutton, and beef under different consumption scenarios. HI > 1 indicates potential non-carcinogenic health risk.

Population Group	Chicken	Mutton	Beef
**Low muscle consumers**	1.66	2.07	2.33
**High muscle consumers**	2.45	3.00	3.18
**Low liver consumers**	0.43	0.88	0.86
**High liver consumers**	1.46	2.25	2.26

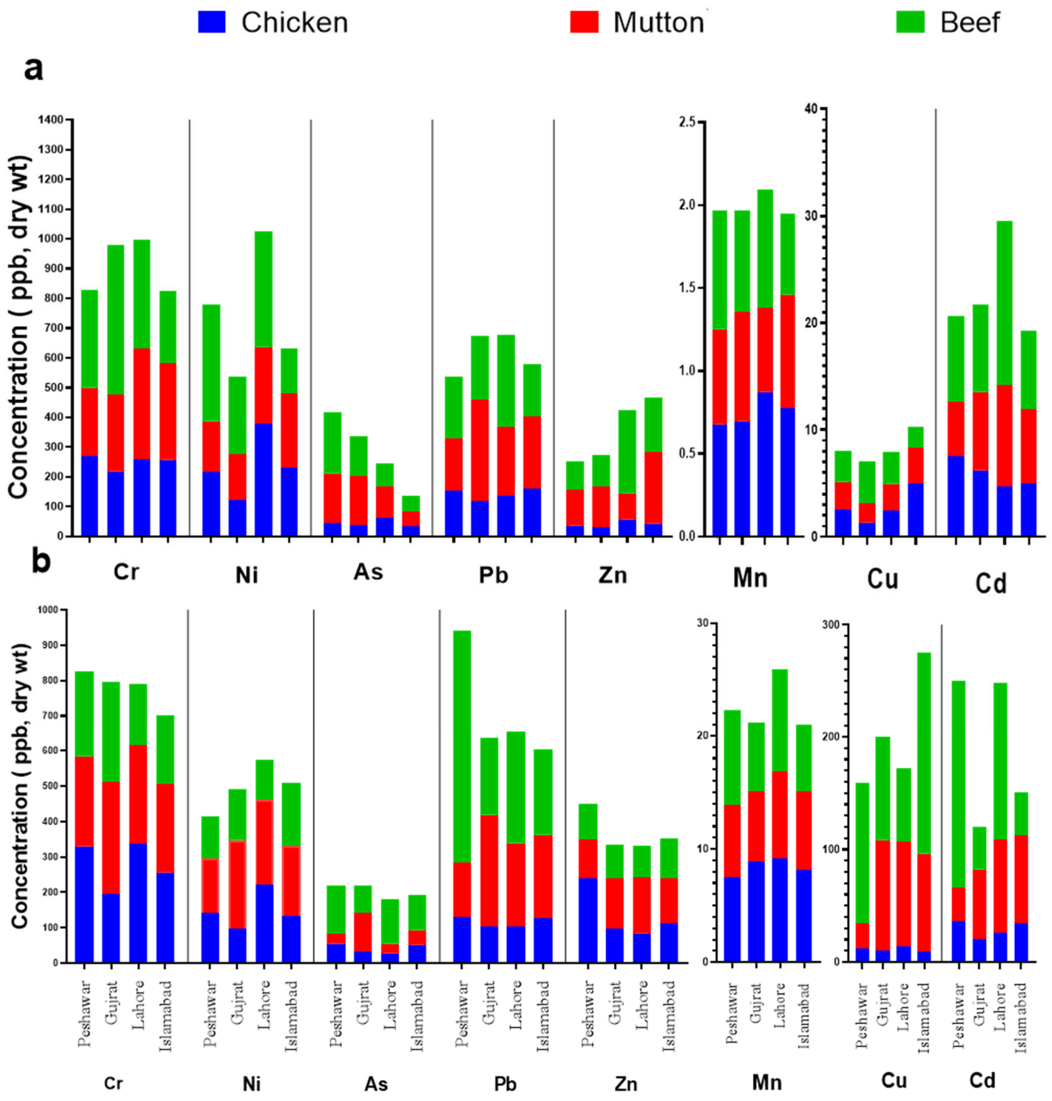

Figure S1 Concentration (μg/kg, dry weight) of PTE in (a) Muscle and (b) Liver tissues within meat in different cities of Pakistan.

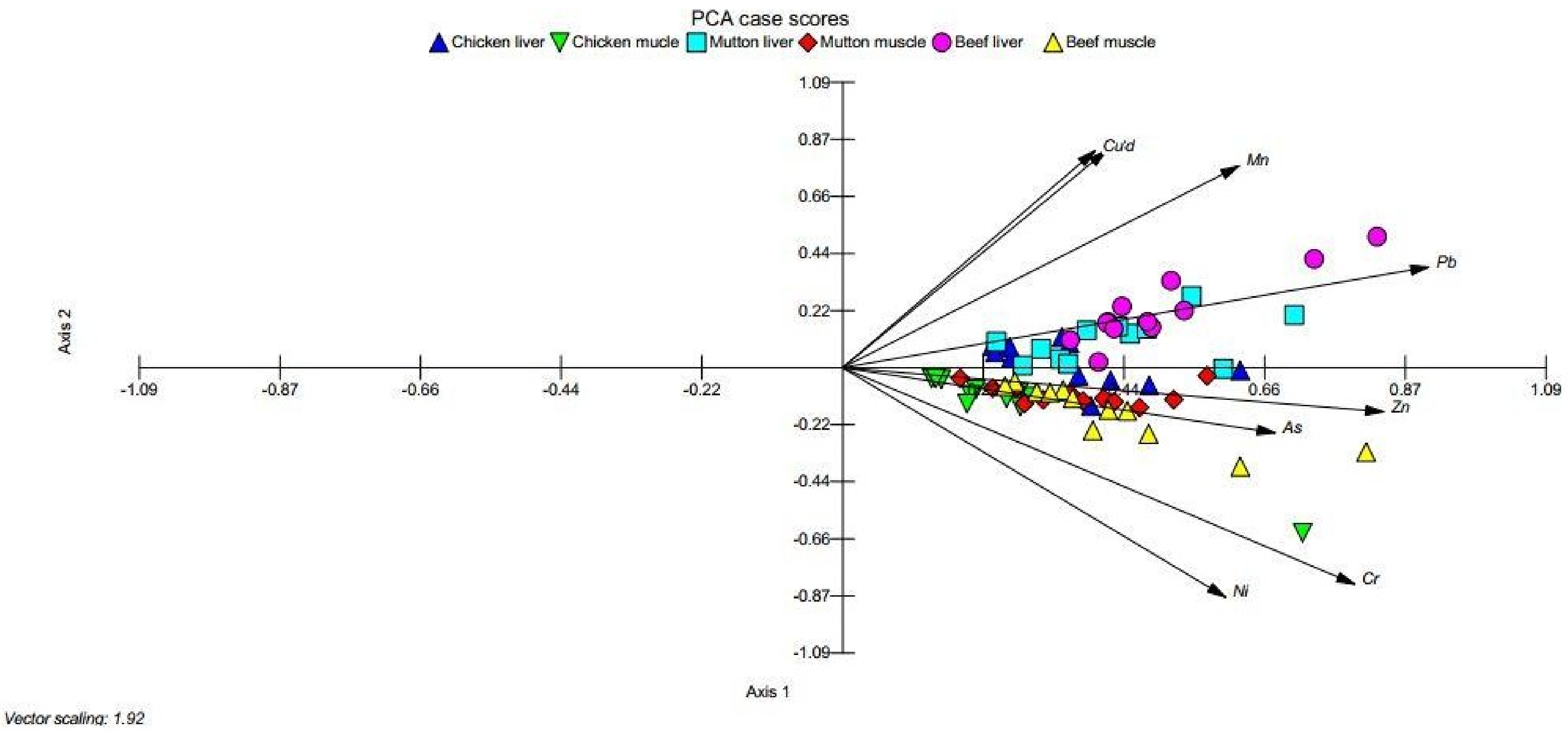

Figure S2 PCA analysis for studied PTEs measured on liver and muscle of different animals.

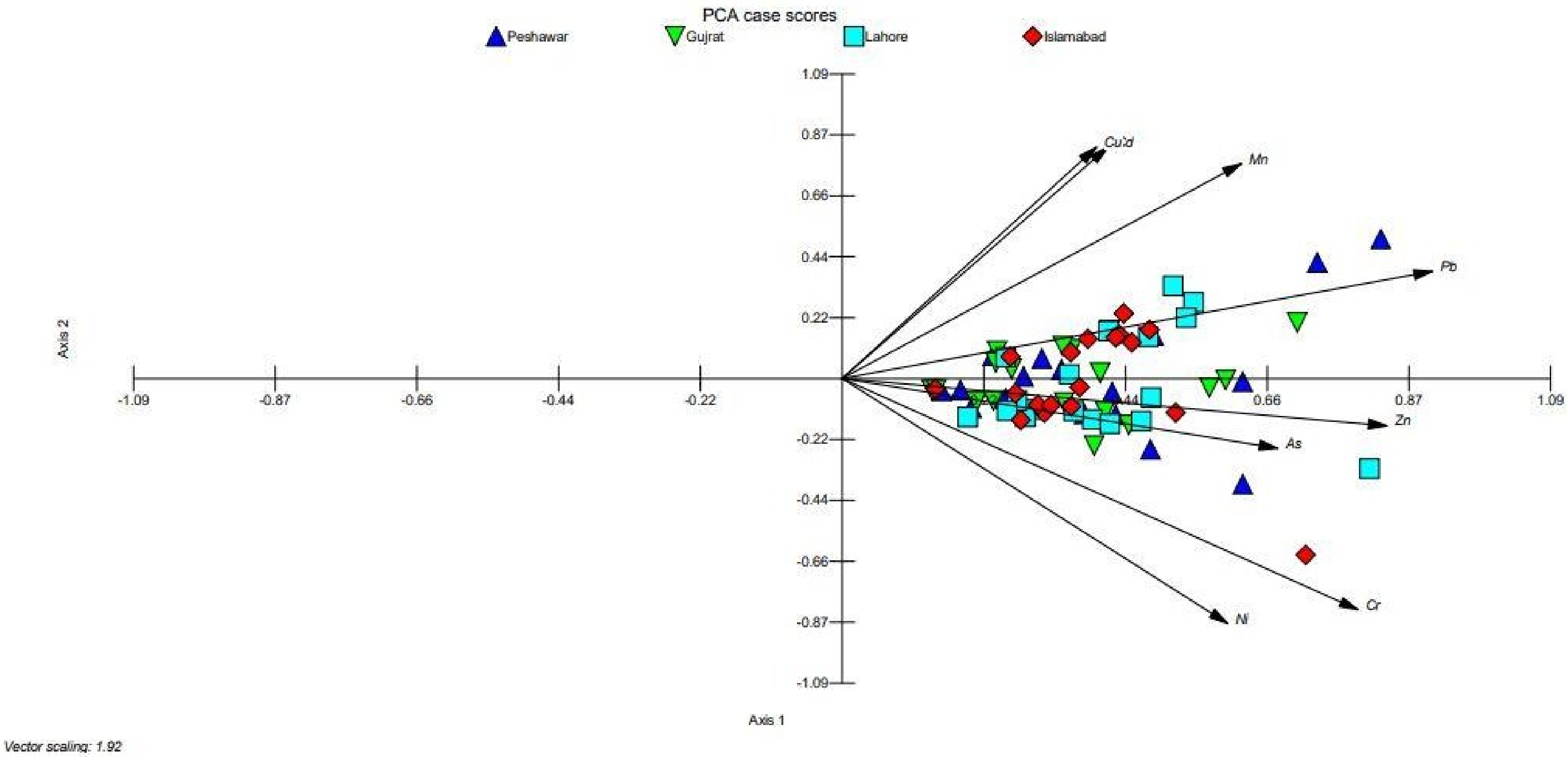

Figure S3 PCA analysis for studied PTEs measured in the studied different animals at different cities of Pakistan.

References

1- Khan, Z., Sultan, A., Khan, R. and Khan, S., 2016. Imranullah and Kamran Farid (2016). Concentrations of heavy metals and minerals in poultry eggs and meat produced in Khyber Pakhtunkhwa, Pakistan. Meat Sci. Vet. Public Heal, 1(1), pp.4-10.

2- Abbas, M., Chand, N., Khan, R.U., Ahmad, N., Pervez, U. and Naz, S., 2019. Public health risk of heavy metal residues in meat and edible organs of broiler in an intensive production system of a region in Pakistan. Environmental Science and Pollution Research, 26(22), pp.23002-23009.

3- Han, J.L., Pan, X.D. and Chen, Q., 2022. Distribution and safety assessment of heavy metals in fresh meat from Zhejiang, China. Scientific Reports, 12(1), p.3241.

4- Khalafalla, F.A., Ali, F.H., Hassan, A.R.H. and Basta, S.E., 2016. Residues of lead, cadmium, mercury and tin in canned meat products from Egypt: an emphasis on permissible limits and sources of contamination. Journal für Verbraucherschutz und Lebensmittelsicherheit, 11(2), pp.137-143.

5- Hamasalim, H.J. and Mohammed, H.N., 2013. Determination of heavy metals in exposed corned beef and chicken luncheon that sold in Sulaymaniah markets. Afr J Food Sci, 7(7), pp.178-82.

6- Imran, R., Hamid, A. and Amjad, R., 2015. Estimation of the heavy metal concentration in the poultry meat being produced in Kasur. J. Bio. and Env. Sci, 7(4), pp.62-75.

7- Akan, J.C., F.I. Abdulrahman, O.A. Sodipo andY.A. Chiroma. 2010. Distribution of Heavy Met-als in the Liver, Kidney and Meat of Beef, Mutton, Caprine and Chicken from Kasuwan ShanuMarket in Maiduguri Metropolis, Borno State,Nigeria. Res. J. Appl. Sci. Engin. Technol. 2(8):743-748.

8- Jankeaw, M., Tongphanpharn, N., Khomrat, R., Iwai, C.B. and Pakvilai, N., 2015. Heavy metal contamination in meat and Crustaceans products from Thailand local markets. International Journal of Environmental and Rural Development, 6(2), pp.153-158.

9- Milam, C., Dimas, B.J., Jang, A.L. and Eneche, J.E., 2015. Determination of some heavy metals in vital organs of cows and bulls at Jimeta Abattoir, Yola, Adamawa State, Nigeria. American Chemical Science Journal, 8(4), pp.1-7.

10- Yabe, J., Nakayama, S.M., Ikenaka, Y., Muzandu, K., Ishizuka, M. and Umemura, T., 2012. Accumulation of metals in the liver and kidneys of cattle from agricultural areas in Lusaka, Zambia. Journal of Veterinary Medical Science, 74(10), pp.1345-1347.

11- Koréneková, B., Skalická, M. and Nad, P., 2002. Concentration of some heavy metals in cattle reared in the vicinity of a metallurgic industry. Veterinarski arhiv, 72(5), pp.259-268.

12- Nkansah, M.A. and Ansah, J.K., 2014. Determination of Cd, Hg, as, Cr and Pb levels in meat from the Kumasi central abattoir. International journal of scientific and research publications, 4(8), pp.1-4.

13- Alkmim Filho, J.F., Germano, A., Dibai, W.L.S., Vargas, E.A. and Melo, M.M., 2014. Heavy metals investigation in bovine tissues in Brazil. Food Science and Technology, 34, pp.110-115.

14- Hoha, G.V., Costăchescu, E., Leahu, A. and Păsărin, B., 2014. Heavy metals contamination levels in processed meat marketed in Romania. Environmental Engineering and Management Journal, 13(9), pp.2411-2415.

15- Nawaz, R., Rehman, S.U., Nawaz, S. and Iftikhar, B., 2015. Analysis of heavy metals in red meat in district Peshawar Khyber Pakhtunkhwa. Journal of Medical Sciences, 23(3), pp.166-171.

16- Khalafalla, F.A., Ali, F.H., Schwagele, F. and Abd-El-Wahab, M.A., 2011. Heavy metal residues in beef carcasses in Beni-Suef abattoir, Egypt. Veterinaria italiana, 47(3), pp.351-361.

17- Miranda, M., López Alonso, M., Castillo, C., Hernández, J. and Benedito, J.L., 2001. Cadmium levels in liver, kidney and meat in calves from Asturias (North Spain). European Food Research and Technology, 212(4), pp.426-430.
